# The Medical Department of the University of Buffalo

**Published:** 1890-10

**Authors:** 


### THE MEDICAL DEPARTMENT OF THE UNIVERSITY OF BUFFALO.

THE opening exercises of the Medical Department of the University of Buffalo were held Monday evening, September 22d, the introductory address having been given by Prof. Ernest Wende. Some changes have been made in the curriculum, such as the introduction of daily clinics at the General Hospital and ward classes, for the clinical study of disease at the bedside. During three days in the week lectures will begin at 9 A. M., instead of 10 A. M., as heretofore. Laboratory practice has been added to, and the dissecting rooms equipped with new facilities for work. They will be open during the evening from 7 to 10 P. M. The number of matriculants is very large, and all-in-all the present session promises to be a successful one in every respect.

